# A Mouse Model of Early-Onset Renal Failure Due to a Xanthine Dehydrogenase Nonsense Mutation

**DOI:** 10.1371/journal.pone.0045217

**Published:** 2012-09-14

**Authors:** Sian E. Piret, Christopher T. Esapa, Caroline M. Gorvin, Rosie Head, Nellie Y. Loh, Olivier Devuyst, Gethin Thomas, Steve D. M. Brown, Matthew Brown, Peter Croucher, Roger Cox, Rajesh V. Thakker

**Affiliations:** 1 Nuffield Department of Clinical Medicine, Oxford Centre for Diabetes, Endocrinology and Metabolism, University of Oxford, Oxford, United Kingdom; 2 Mammalian Genetics Unit, MRC Harwell, Harwell Science and Innovation Campus, United Kingdom; 3 Institute of Physiology, Zurich Center for Integrative Human Physiology, University of Zurich, Zurich, Switzerland; 4 The University of Queensland Diamantina Institute, Princess Alexandra Hospital, Woolloongabba, Queensland, Australia; 5 Garvan Institute for Medical Research, Sydney, Australia; National Cancer Institute, United States of America

## Abstract

Chronic kidney disease (CKD) is characterized by renal fibrosis that can lead to end-stage renal failure, and studies have supported a strong genetic influence on the risk of developing CKD. However, investigations of the underlying molecular mechanisms are hampered by the lack of suitable hereditary models in animals. We therefore sought to establish hereditary mouse models for CKD and renal fibrosis by investigating mice treated with the chemical mutagen *N*-ethyl-*N*-nitrosourea, and identified a mouse with autosomal recessive renal failure, designated RENF. Three-week old RENF mice were smaller than their littermates, whereas at birth they had been of similar size. RENF mice, at 4-weeks of age, had elevated concentrations of plasma urea and creatinine, indicating renal failure, which was associated with small and irregularly shaped kidneys. Genetic studies using DNA from 10 affected mice and 91 single nucleotide polymorphisms mapped the *Renf* locus to a 5.8Mbp region on chromosome 17E1.3. DNA sequencing of the xanthine dehydrogenase (*Xdh*) gene revealed a nonsense mutation at codon 26 that co-segregated with affected RENF mice. The *Xdh* mutation resulted in loss of hepatic XDH and renal Cyclooxygenase-2 (COX-2) expression. *XDH* mutations in man cause xanthinuria with undetectable plasma uric acid levels and three RENF mice had plasma uric acid levels below the limit of detection. Histological analysis of RENF kidney sections revealed abnormal arrangement of glomeruli, intratubular casts, cellular infiltration in the interstitial space, and interstitial fibrosis. TUNEL analysis of RENF kidney sections showed extensive apoptosis predominantly affecting the tubules. Thus, we have established a mouse model for autosomal recessive early-onset renal failure due to a nonsense mutation in *Xdh* that is a model for xanthinuria in man. This mouse model could help to increase our understanding of the molecular mechanisms associated with renal fibrosis and the specific roles of XDH and uric acid.

## Introduction

Chronic kidney disease (CKD) is a major health problem worldwide. Due to its progressive nature, patients with CKD are at risk of developing cardiovascular disease and end stage renal failure (ESRF), often in association with secondary hyperparathyroidism. Regardless of the initial cause, CKD and ESRF are invariably associated with renal fibrosis, and often with glomerulosclerosis, and immune activation, which leads to renal failure [1]. The development of fibrosis within the kidney is a major factor in renal impairment, although the mechanisms underlying this are not fully understood and there are no effective treatments available to retard or reverse its progression [1]. Population and genome-wide association studies (GWAS) carried out in different ethnic populations have suggested a genetic component in the susceptibility to developing renal failure. For example, single nucleotide polymorphisms (SNPs) within the myosin heavy chain type II isoform A (*MYH9*)-Apolipoprotein L1 (*APOL1*) gene region were found to be associated with ESRF in African Americans [2], [3], whilst several associations have been identified between SNPs and CKD or markers of decreased renal function, such as SNPs near genes involved in nephrogenesis, e.g. *ALMS1*, or solute transport, e.g. *SLC7A9*, that were associated with CKD [4]. Furthermore, some genes identified as associated with CKD in the general population by GWAS, such as *UMOD* that encodes uromodulin, can also cause monogenic renal disorders due to rare pathogenic mutations [4], [5]; thus both GWAS and studies of monogenic inherited diseases can help to elucidate important biological pathways. However, further investigations of the underlying genetic and molecular mechanisms of CDK have been hampered by the lack of suitable hereditary models in animals. To facilitate such studies, we embarked on establishing mouse models for renal fibrosis and CKD by investigating the phenotypes of progeny of mice treated with the chemical mutagen *N*-ethyl-*N*-nitrosourea (ENU). ENU is a potent mutagen in mice, and as an alkylating agent, introduces mainly point mutations into the genome [6]. Phenotype-driven screens of the offspring of ENU-mutagenised mice are hypothesis-generating, and utilise a panel of morphological, biochemical and other tests to identify phenotypes of interest [6]. Since phenotype-driven screens do not make any assumptions about the genetic cause of a phenotype, they may reveal novel genes and pathways not previously associated with the phenotype. ENU mutagenesis has generated a variety of mouse models, including dominant and recessive, hypo- and hypermorphs, for different diseases including renal disorders, such as Catweasel mice, which are a model for branchio-oto-renal syndrome due to a hypomorphic mutation in *Six1* [7]. Here, we report studies of one of the models which was identified during an ENU screen, designated RENF (Renal Failure), which is a mouse model for autosomal recessive early-onset renal failure.

## Results

### Phenotypic Identification of RENF Mice

**Figure 1:**
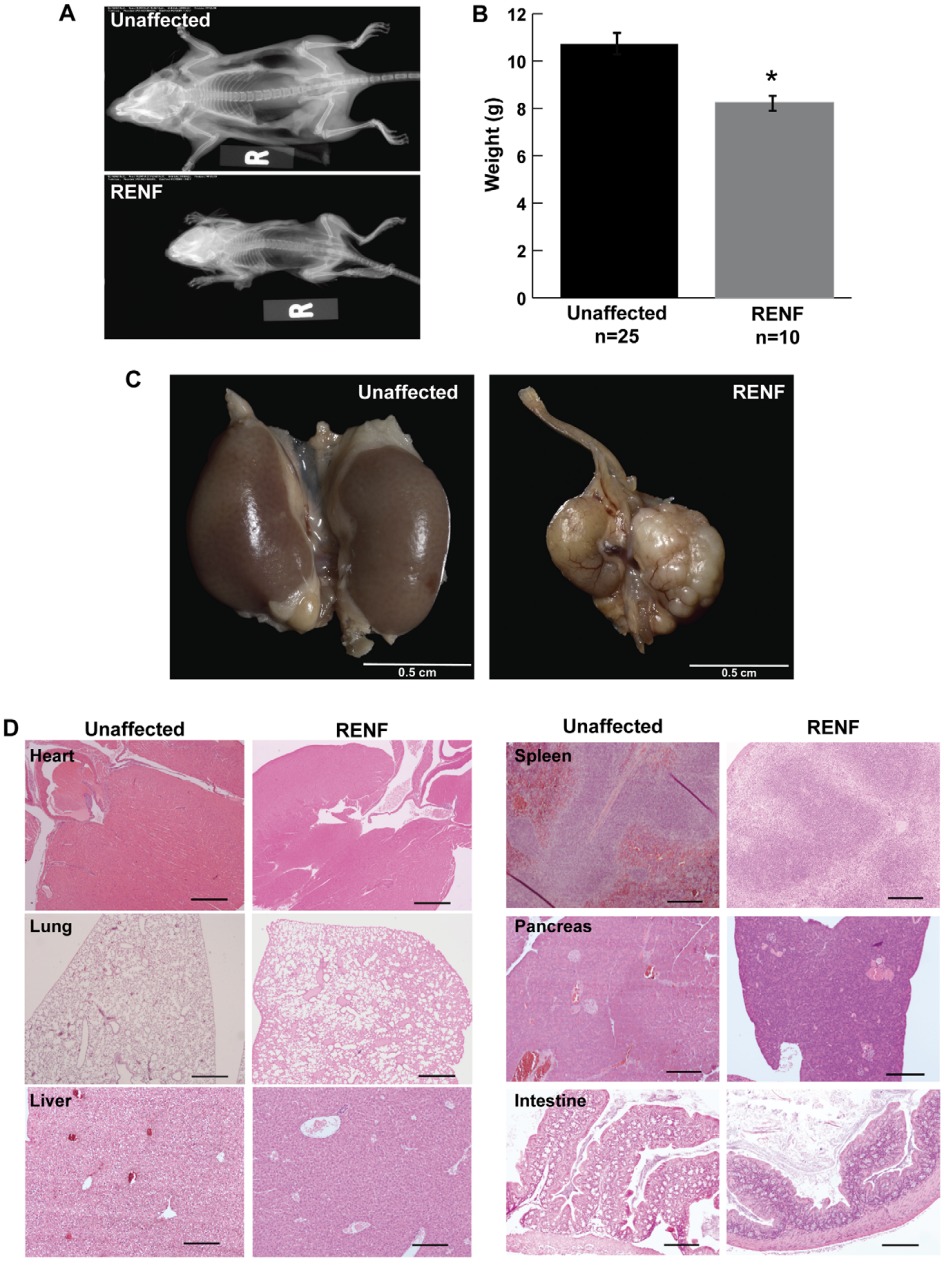
Gross anatomical features of RENF mice. (A) Whole-body X-ray of 3-week old mice revealing small size of RENF mice. (B) Weights of 3-week old mice revealing reduced weight of RENF mice, when compared to unaffected littermates (* = p<0.001). (C) Anatomical appearance of kidneys from 4-week old mice revealing smaller, irregularly shaped and fatty kidneys from a RENF mouse compared to an unaffected littermate. (D) Histological appearance of other organs stained by H&E from 4-week old mice, revealing normal anatomy in unaffected and affected RENF mice. Scale bars: 200µm for heart and lung; 50µm for liver, spleen, pancreas and intestine.

The detection of abnormal renal function in G3 mice was based on elevated plasma creatinine and urea concentrations, and/or abnormal gross renal morphology. RENF mice, at 3 weeks of age, were smaller (Figure 1A) and weighed ∼23% less than their unaffected littermates (10.72±0.33g for unaffected mice versus 8.27±0.31g for affected mice, p<0.001) (Figure 1B), whereas at birth, RENF mice and their unaffected littermates had been of the same size. They failed to thrive and did not live beyond 5–9 weeks of age. Post mortem analysis undertaken at 4 weeks of age revealed that kidneys from RENF mice were smaller, irregularly shaped and had a fatty appearance when compared to kidneys from unaffected littermates (Figure 1C). The anatomy of other internal organs appeared normal (Figure 1D). Inheritance testing could not be undertaken through mating of the mutant mice because they died before puberty. However, random intercrossing of unaffected male and female littermates resulted in progeny in which ∼25% had the RENF phenotype, consistent with autosomal recessive inheritance.

### Abnormal Plasma Biochemical Profile in RENF Mice

Biochemical analysis of plasma at 4 weeks of age revealed male RENF mice to have elevated levels of several biochemical parameters, when compared to unaffected mice. Thus, RENF mice had significantly elevated concentrations of urea and creatinine (p<0.005) (Table 1), indicating renal failure. Moreover, two RENF mice had elevated plasma urea concentrations that were above the upper limit of detection and could not be included in the analysis. In addition, RENF mice had increased concentrations of calcium (corrected for albumin) (p<0.01) and increased alkaline phosphatase activity (p<0.05) (Table 1), consistent with tertiary hyperparathyroidism in RENF mice.

**Table 1:** Plasma biochemical analysis of 4-week old male mice.

	Unaffected (n = 4)	RENF (n = 5)	P-value^a^
**Creatinine (µmol/l)**	42.33±11.86	187.26±18.72	<0.005
**Urea (mmol/l)**	10.18±2.50	80.90±11.82^b^	<0.005
**Corr.Ca (mmol/l)**	2.65±0.06	3.13±0.10	<0.01
**Phosphorous (mmol/l)**	4.00±0.66	6.51±0.92	= 0.07
**ALP (U/l)**	236.50±92.00	682.40±137.84	<0.05

Corr.Ca: calcium concentration corrected for albumin, derived from the equation Corr.Ca = Ca (mmol/l)−((Alb(g/l)−30)×0.017); ALP: alkaline phosphatase.

a p-values calculated using Student’s unpaired, two-tailed t-test.

b n = 3 as two samples had a urea concentration greater than the upper limit of the assay.

### Mapping of the *Renf* Locus to Chromosome 17E1.3 and Identification of a *Xdh* Nonsense Mutation

**Figure 2 pone-0045217-g002:**
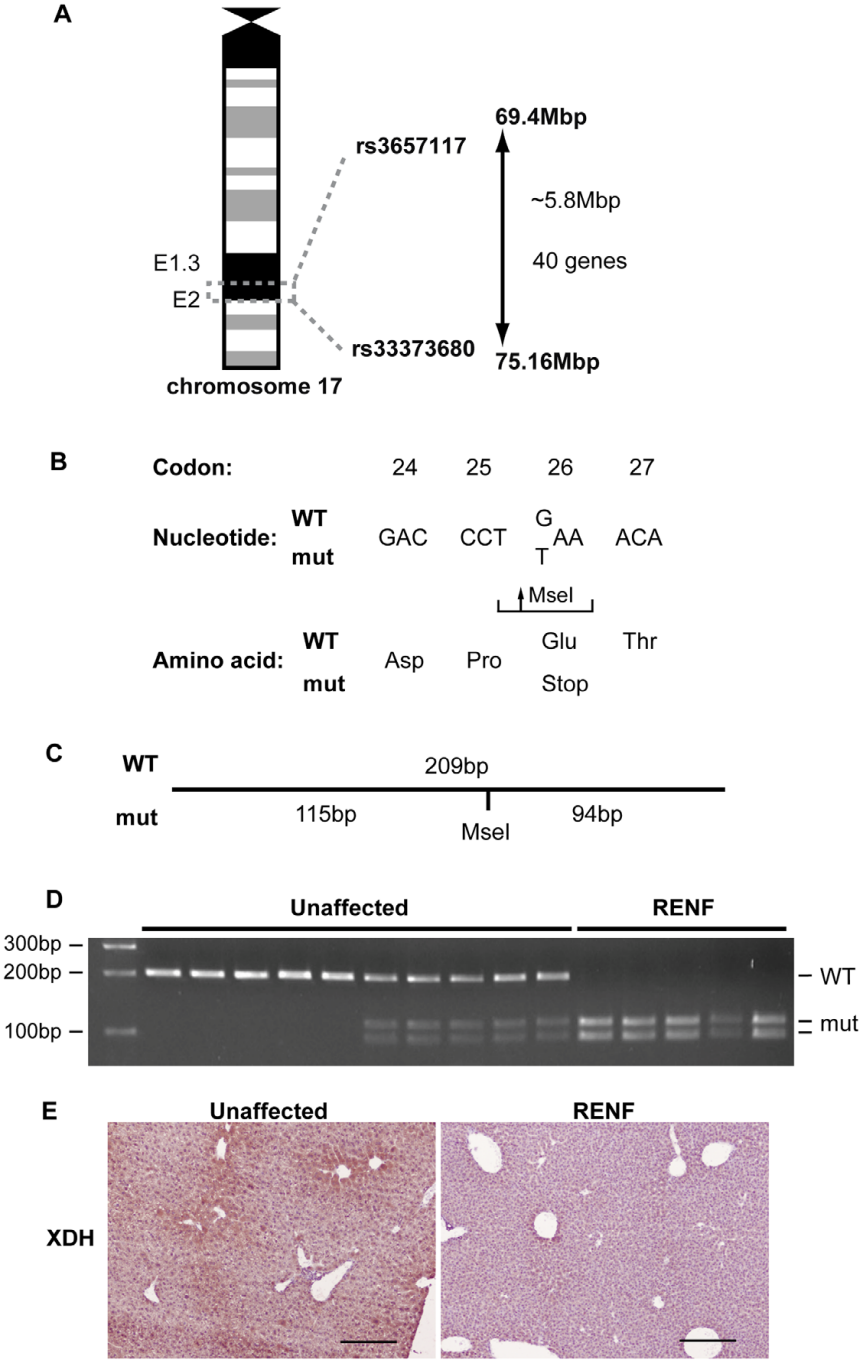
Mapping and identification of the gene mutation causing RENF. (A) The RENF locus was mapped to a 5.8Mbp interval flanked by the SNPs rs3657117 and rs33373680 on chromosome 17, which contained 40 genes. (B) DNA sequence analysis of the *Xdh* gene revealed a homozygous G>T transversion in exon 2, changing codon 26 from GAA to TAA, resulting in a nonsense mutation. (C) The Glu26Stop mutation also generated a MseI restriction endonuclease recognition site, thus cleavage of a 209 bp PCR product by MseI yielded 115bp and 94bp fragments in mutant alleles but did not cut wild-type alleles. (D) MseI digest was used to genotype unaffected and affected RENF mice. All affected RENF mice were homozygous for the Glu26Stop mutation. (E) Immunohistochemistry showed loss of hepatic XDH expression in RENF mice compared to unaffected littermates. Scale bars = 200 µm.

Genome-wide analysis using 91 SNPs and DNA from 10 affected and 14 unaffected mice mapped the *Renf* locus to a 5.8Mb region on chromosome 17 flanked by the SNPs rs3657117 and rs33373680 (Figure 2A). This interval contained 40 genes (Table 2), of which 23 have been reported to be expressed in the kidney. Among these 23 genes, only xanthine dehydrogenase (*Xdh*) is known to cause autosomal recessive renal failure in man due to loss of function mutations [8]–[12]. Mutational analysis of the *Xdh* gene was therefore undertaken in RENF mice by DNA sequencing, which revealed a homozygous G>T transversion causing a nonsense mutation at codon 26, Glu26Stop (E26X) (Figure 2B). This mutation resulted in a gain of an MseI restriction endonuclease recognition site in exon 2 of *Xdh* (Figure 2B,C), which was utilised for genotyping of unaffected and affected RENF mice. All affected mice were homozygous for the E26X mutation (Figure 2D). Re-analysis of the weights of unaffected mice showed no difference between the weights of wild-type and heterozygous mice at weaning (data not shown). The *Xdh* gene encodes the enzyme, XDH, that catalyses the predominantly hepatic oxidative conversion of xanthine and hypoxanthine to uric acid in the purine catabolic pathway. Immunohistochemical analysis of hepatic sections from RENF mice and wild-type littermates revealed that the *Xdh* E26X mutation in the RENF mice resulted in loss of hepatic XDH expression (Figure 2E). Mouse *Xdh* consists of 36 exons, which encode 1335 amino acids with 89% identity and 95% similarity with the human XDH protein. Loss-of-function mutations of *XDH* in man cause hereditary xanthinuria, which is characterised by very low or undetectable plasma uric acid levels [8]–[12]. We therefore measured plasma uric acid levels in a subset of unaffected and affected RENF mice, and found that in 3 out of 3 RENF mice, the uric acid concentration was below the limit of detection for the assay, compared to concentrations of 72 µmol/l, 107 µmol/l and 116 µmol/l in 3 unaffected mice.

**Table 2:** Genes within the 5.8Mbp interval containing the *Renf* locus on Chromosome 17, and their level of renal expression.

Gene name (symbol)	Renal expression
Erythrocyte protein band 4.1-like 3 (*Epb4.1l3*)	++
Zinc finger protein 161 (*Zfp161*)	+
RIKEN cDNA C030034I22 gene (*C030034I22Rik*)	–
RIKEN cDNA A330050F15 gene (*A330050F15Rik*)	–
Discs, large (Drosophila) homolog-associated protein 1 (*Dlgap1*)	–
TGFB-induced factor homeobox 1 (*Tgif1*)	+
Myosin, light chain 12B, regulatory (*Myl12b*)	–
Myosin, light chain 12A, regulatory, non-sarcomeric (*Myl12a*)	+
Myomesin 1 (*Myom1*)	–
Lipin 2 (*Lpin2*)	+
Elastin microfibril interface 2 (*Emilin2*)	–
SMC hinge-domain containing 1 (*Smchd1*)	+
NDC80 homolog, kinetochore complex component (S. cerevisiae) (*Ndc80*)	–
Methyltransferase like 4, pseudogene 1 (*Mettl4-ps1*)	–
Speedy homolog A (Xenopus laevis) (*Spdya*)	–
tRNA methyltransferase 61 homolog B (S. cerevisiae) (*Trmt61b*)	+
WD repeat domain 43 (*Wdr43*)	++
Small nucleolar RNA, C/D box 92 (*Snord92*)	NK
Small nucleolar RNA, C/D box 53 (*Snord53*)	NK
Family with sequence similarity 179, member A (*Fam179a*)	–
cDNA sequence BC027072 (*BC027072*)	–
CAP-GLY domain containing linker protein family, member 4 (*Clip4*)	+
Anaplastic lymphoma kinase (*Alk*)	–
Yippee-like 5 (Drosophila) (*Ypel5*)	++
Limb-bud and heart (*Lbh*)	+
Lysocardiolipin acyltransferase 1 (*Lclat1*)	++
Calpain 13 (*Capn13*)	–
UDP-N-acetyl-alpha-D-galactosamine:polypeptide N-acetylgalactosaminyltransferase 14 (*Galnt14*)	+
EH-domain containing 3 (*Ehd3*)	+
Xanthine dehydrogenase (*Xdh*)	++
Steroid 5 alpha-reductase 2 (*Srd5a2*)	+
Mediator of cell motility 1 (*Memo1*)	+
Dpy-30 homolog (C. elegans) (*Dpy30*)	++
Spastin (*Spast*)	+
Solute carrier family 30 (zinc transporter), member 6 (*Slc30a6*)	++
NLR family, CARD domain containing 4 (*Nlrc4*)	NK
Ribosomal protein S6 pseudogene (*Gm6476*)	+
Yip1 domain family, member 4	NK
Baculoviral IAP repeat-containing 6 (*Birc6*)	+
Tetratricopeptide repeat domain 27 (*Ttc27*)	+

TGFB: transforming growth factor-β; SMC: structural maintenance of chromosomes; CAP-GLY: cytoskeleton-associated proteins-glycine rich; EH: Eps15 homology; NLR: Nod-like receptor; CARD: caspase activation and recruitment domain; IAP: inhibitor of apoptosis. – no expression; + weak expression; ++ moderate expression. NK: not known. Expression data obtained from BioGPS [32].

### Gross Morphological and Cellular Lesions in RENF Kidneys

**Figure 3:**
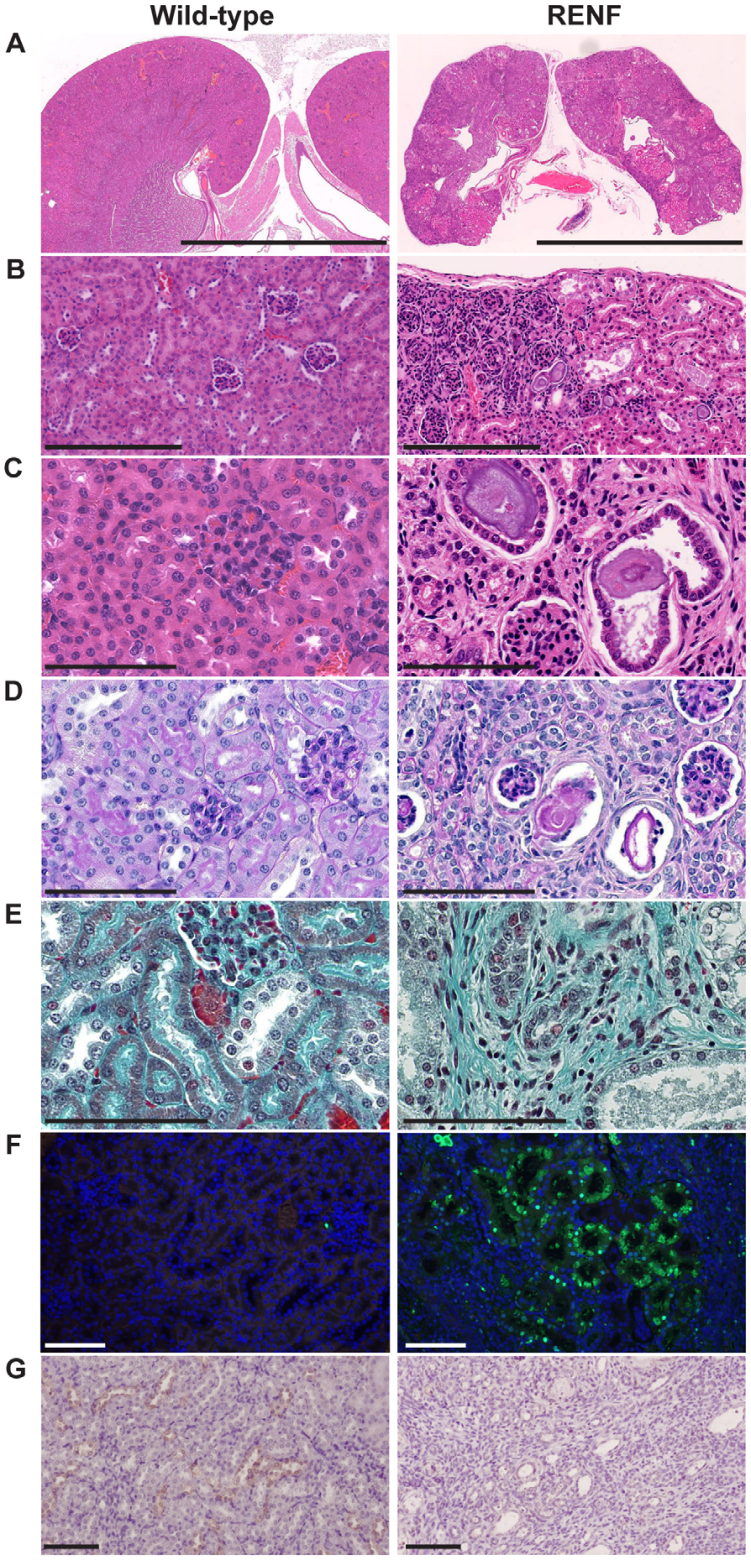
Histological analysis of kidney sections from 4 week old RENF and unaffected mice. (A–C) H&E staining showing: (A) small size and grossly abnormal morphology of RENF kidneys (scale = 5mm); (B) condensed renal cortex with some areas containing a high density of glomeruli in RENF kidneys compared to even distribution of glomeruli and tubules in unaffected kidneys (scale = 250µm); and (C), luminal casts within dilated tubules that are detached from the basement membrane in RENF kidneys (scale = 100µm). (D) Periodic-acid Schiff (PAS) staining showing PAS-positive intraluminal casts surrounded by nuclei, and thickened basement membranes in RENF kidneys (scale = 100µm). (E) Masson’s trichrome staining showing the presence of fibrosis in RENF mice (blue) when compared to unaffected littermates (scale = 100µm). (F) TUNEL staining showing apoptosis of tubular epithelial cells in kidneys from RENF mice, which was not detected in kidneys from unaffected littermates (scale = 100µm). (G) Immunohistochemical analysis of COX-2 expression showed loss of COX-2 expression in affected RENF mice compared to unaffected littermates (scale = 50µm).

Histological analysis of kidney sections using H&E staining confirmed the smaller size of kidneys from RENF mice and also revealed an irregular shape of the outer kidney capsule in RENF mice, when compared to kidneys from unaffected littermates (Figure 3A). There was an altered distribution of glomeruli within the kidneys of RENF mice compared to wild-type littermates, thus in wild-type mice, glomeruli were distributed around the renal cortex, but in RENF mice, there was a high density of glomeruli in some regions, with other regions being almost devoid of glomeruli and having only tubules (Figure 3B). The areas of dense glomeruli corresponded to dips in the outer capsule, and the areas of tubules corresponded to bulges in the outer capsule (Figure 3A,B). There was extensive tubular cell damage with detachment of cells within dilated tubules, and accumulation of eosinophilic amorphous intraluminal casts in kidneys from RENF mice, in some cases filling the cross-sectional area of the dilated tubule (Figure 3C), and some damaged tubules were detached from the basement membrane. There was also extensive lymphocytic infiltration in RENF kidneys (Figure 3B,C), which was absent in wild-type kidneys. The intratubular aggregates were periodic-acid Schiff (PAS)-positive and some were surrounded by nuclei. PAS staining also revealed thickening and shrinkage of the tubular basement membrane (Figure 3D). Staining of RENF and normal kidney sections with von Kossa was negative for calcium mineralization and Congo red staining did not reveal any deposition of amyloid in RENF mice (data not shown). Staining using Masson’s trichrome revealed a disorganized pattern of collagen and reticular fibres in kidneys from RENF mice compared to unaffected kidneys (Figure 3E), indicating the presence of interstitial fibrosis. TUNEL analysis of kidney sections revealed extensive apoptosis of tubular epithelial cells in RENF mice when compared to unaffected littermates (Figure 3F). Cyclooxygenase-2 (COX-2) expression, which is required for post-natal renal development and has previously been shown to be lost in mice deficient for xanthine oxidoreductase (*Xor* ^−/−^) [13], was found to be virtually absent in kidney sections of RENF mice when compared to those from unaffected littermates, in whom COX-2 expression was detected in a subset of tubules predominantly in the corticomedullary region (Figure 3G).

## Discussion

In this study we describe an ENU-induced mouse mutant named RENF, presenting with clinical features of failure to thrive, early onset renal failure and interstitial nephritis, due to a *Xdh* nonsense mutation which is located within the iron-sulphur (2Fe-2S) ferredoxin-type domain close to the N-terminus of XDH (Table 1, Figures 1–3). This E26X mutation (Figure 2B, C and D) was induced by ENU, which is known to induce multiple mutations simultaneously [14]. However, the likelihood that another genetic defect within the 5.8Mbp region that was established to be the location of the *Renf* locus (Figure 2A), could be the underlying cause of RENF is <0.005, based on the following reasoning. The nominal ENU-induced base pair mutation rate for potentially functional mutations has been estimated to be 1 in 1.82Mbp of coding DNA in the F1 founder animals [15], and given that <2.5% of the mouse genome is coding, it has been calculated that the probability of two functional mutations arising within a 5Mb genomic region is <0.002 [16]; thus, the likelihood of the *Xdh* E26X mutation and another functional mutation arising within the 5.8Mbp containing the *RENF* locus is <0.0023. This indicates that the *Xdh* E26X mutation, which was shown to be associated with loss of hepatic XDH (Figure 2E) and renal COX-2 expression (Figure 3G), is highly likely to be the sole genetic defect causing RENF.

RENF mice have phenotypic characteristics that are similar to those reported for the *Xor* ^−/−^ mouse, including early mortality, renal failure, interstitial nephritis with intratubular deposits, and undetectable plasma uric acid (Table 3). These similarities between the RENF and *Xor* ^−/−^ mice are consistent with the loss of expression of XDH (Figure 2E) and COX-2 (Figure 3G) in both mouse models. However, RENF mice had extensive apoptosis of tubular cells (Figure 3F), and elevated plasma calcium and ALP (Table 1), consistent with tertiary hyperparathyroidism, as well as elevated plasma creatinine concentrations, which were not reported in the *Xor* ^−/−^ mice [13], whereas *Xor* ^−/−^ mice had renal cysts (Table 3), which were not detected in RENF mice. The basis of these differences between the RENF mice and *Xor* ^−/−^ mice remains to be elucidated. A possible explanation may involve strain-specific differences, as the *Xor* ^−/−^ mice were on a mixed 129/Sv–C57BL/6J background [13], whilst the RENF mice were on a C3H–C57BL/6J mixed background. In addition, ENU-induced mouse models have been reported to differ in phenotypic features when compared to the corresponding null mice, generated using targeted gene ablation strategies [14]. For example, mice with a hypomorphic ENU mutation in the forkhead transcription factor *Foxc1* have been reported to have phenotypic differences when compared to mice deficient for *Foxc1*; thus, the ENU hypomorphic mice, unlike the *Foxc1* deficient mice, did not die *in utero*, thereby facilitating the study of previously uncharacterised roles for FOXC1. In addition, the ENU hypomorphic *Foxc1* mice, when compared to *Foxc1* deficient mice, had greater similarity to patients with Axenfeld-Rieger syndrome (ARS) that is due to *Foxc1* mutations [17]. These differences between the ENU-induced RENF and *Xor* ^−/−^ mouse models highlight the advantages of having two allelic variants that provide the opportunity of elucidating further the relationships between the phenotype and the likely protein structure and its *in vivo* function [18].

**Table 3:** Comparison of XOR^−/−^ and RENF mouse models.

Characteristic	XOR^−/−^ mice^a^	RENF mice
Derivation of mutation	Gene disruption	ENU
Nature of mutation	KO	Nonsense
Renal failure	+	+
Early death	+	+
Plasma urea	↑	↑
Plasma creatinine	Normal	↑
Plasma corrected calcium	NR	↑
Plasma ALP	NR	↑
Plasma uric acid	Below detection limit	Undetectable
Renal cysts	+	–
Deposits in renal tubules	+	+
Inflammatory infiltration	+	+
Renal interstitial fibrosis	+	+
Renal tubular apoptosis	NR	↑

+, present; –, absent; ↑, increased; ↓, decreased; NR, not reported; ND, not determined; ALP, alkaline phosphatase; COX-2, cyclooxygenase-2; KO, knock-out; ENU, *N*-ethyl-*N*-nitrosourea.

a Data for xanthine oxidoreductase null (XOR^−/−^) mice from Ohtsubo *et al*. 2004 [13] and Ohtsubo *et al*. 2009 [33].

Mutations in XDH in man give rise to classical xanthinuria type I, a rare genetic disorder characterised by elevated serum and urinary concentration of xanthine and renal xanthine calculi, in association with undetectable levels of uric acid [8]–[12]. Mutations detected in man have included nonsense mutations at codons 228 and 722 [8], [11], frameshifting insertions and deletions [8], [9], [12], and missense mutations [10], [12]. One patient with a nonsense mutation (R228X) was found to have an absence of XDH protein [8], whilst assays of XDH activity in duodenal mucosa biopsy samples from patients with a frameshift insertion at codon 569 and a missense mutation (R149C) showed ∼5% and undetectable XDH activities, respectively [9], [10]. Thus, these genetic defects are likely to result in a loss of XDH activity similar to that in *Xor* ^−/−^ and RENF mice (Figure 2E). However, a major difference between xanthinuria in man and the phenotype of both the *Xor* ^−/−^ and RENF mice is the striking renal phenotype in the mouse models, which leads to premature death within several weeks (Table 2). In contrast, patients with xanthinuria do not always progress to renal failure, and some remain asymptomatic [9]. This difference between mouse and man is likely due to post-natal renal development in mice, which does not occur in man. In man, nephrogenesis and glomerulogenesis are completed before birth [19]; however murine post-natal renal development is dependent on induction of COX-2 expression, which is absent in *Xor* ^−/−^ and RENF mice (Figure 3G), although it is not clear whether induction of COX-2 expression is due to uric acid or to another function of XDH [13]. The role of XDH and COX-2 in post-natal renal development in mice is consistent with the lack of any distinguishable abnormalities in the RENF mice at birth and in the *Xor* ^−/−^ or *Cox-2* null mice before 1 week of age [13], [20]; furthermore, the kidneys of *Cox-2* null mice at ages 3 and 7 days have also been reported to be normal [20]. Indeed, the altered distribution of glomeruli in RENF mice (Figure 3B) is suggestive of a developmental defect, and is reminiscent of a similar phenotype described in *Cox-2* null mice, in which glomeruli were clustered in the subcapsular region [20]. In man, renal development is completed before birth, and during gestation, the abnormal purine metabolism in the foetus is likely to be compensated for by maternal XDH, since uric acid, xanthine and hypoxanthine can cross the placental membrane. Whilst the renal symptoms in xanthinuria patients can be attributed directly to the elevated levels of xanthine in the kidney, it is unclear whether the absence of plasma uric acid has any additional detrimental effect. In *Xor* ^−/−^ and RENF mice, the developing foetus can also benefit from maternal XDH activity; however plasma uric acid levels will decrease rapidly in the immediate post-natal period, since the expression of uricase in mice, which is evolutionarily silenced in man, will metabolise any plasma uric acid to allantoin [21]. In contrast, in man, more than 90% of filtered uric acid is reabsorbed by the kidney and not metabolised [21], thus plasma uric acid levels may be maintained for longer after birth, thus any effect of lack of uric acid on the phenotype may be magnified in mice compared to man. Studies of models such as *Xor* ^−/−^ and RENF may help to clarify the relative roles of elevated xanthine and lack of uric acid, in the pathogenesis of xanthinuria, for example by the use of uricase inhibitors to maintain plasma uric acid levels after birth.

Widespread fibrosis, large intratubular deposits, and extensive apoptosis of tubular epithelial cells were observed in kidneys of RENF mice (Figure 3D, E and F). Apoptosis of tubular cells may be caused by mechanical stress as a result of intratubular deposits [22]. However, the mechanism by which apoptotic cells may trigger a cascade of pro-fibrotic events is not clear, but may involve uric acid, which is released by apoptotic cells, and acts as a ‘danger’ signal to stimulate immune cells [23]. Thus, a local high concentration of uric acid may stimulate resident interstitial immune cells, which could then initiate the fibrotic cascade, an effect that may be more potent in the context of the otherwise low systemic concentration of uric acid in the RENF mice. The absence of expression of COX-2 in the RENF mice (Figure 3G) may further contribute to the renal fibrosis as follows. Fibrosis is observed in the *Cox-2* null mice [20], and this likely involves its main substrate, arachidonic acid, which can also be metabolised by 5-lipoxygenase (5-LOX) to generate leukotrienes that are able to stimulate inflammatory processes [24]. Thus, in the absence or reduction of COX-2, the flux through the 5-LOX pathway may be increased, as reported by studies of lungs from *Cox-2* null mice that had an increased inflammatory response to an allergy challenge compared to wild-type mice [25], and by administration of Cox inhibitor in rats that led to an increase in levels of the leukotriene LTB_4_ [24]. These observations suggest that the absence of COX-2 expression in RENF kidneys (Figure 3G) may also propagate any damage that is initiated by the intratubular deposits, by causing inflammation and triggering a fibrotic cascade.

In summary, our studies have established a novel mouse model for *Xdh* deficiency, due to an ENU-induced nonsense mutation, that is associated with early onset renal failure and fibrosis. Our studies demonstrate that ENU mutagenesis may be useful for generation of mouse models for renal failure, and that this *RENF* model may help to elucidate further mechanisms of renal fibrosis and the *in vivo* role of XDH and uric acid.

## Materials and Methods

### Ethics Statement

All animal studies were carried out using guidelines issued by the Medical Research Council in ‘Responsibility in the Use of Animals for Medical Research’ (July 1993) and Home Office Project License Number 30/2433. Experiments were approved by the Medical Research Council Harwell ethics committee, and all efforts were made to minimize suffering.

### Generation of Mutant Mice

Male C57BL/6J mice were treated with ENU and mated with untreated C3H female mice [14]. The male progeny (G1) were subsequently mated with normal C3H females to generate G2 progeny. The female G2 progeny were backcrossed to their G1 fathers and the resulting G3 progeny were screened for recessive phenotypes [18]. Mice were fed an expanded rat and mouse no. 3 breeding diet (Special Diets Services, Witham, UK) containing 1.15% calcium, 0.82% phosphorus and 4088.65 units/kg vitamin D, and given water *ad libitum*.

### Dysmorphology and Biochemical Screening

A simple dysmorphology screen based on observation and weighing of mice to look for any gross anatomical changes was used to monitor all mice as previously described [26]. Blood samples were collected from the retro-orbital sinus after terminal anaesthesia, and plasma was separated by centrifugation at 3000×g for 5 min at 4°C, and analysed for urea, creatinine, total calcium, inorganic phosphate, alkaline phosphatase, albumin and uric acid, on a Beckman Coulter AU680 semi-automated clinical chemistry analyzer using reagents and protocols provided by the manufacturer. Plasma calcium was corrected for albumin (Corr.Ca) using the formula: Corr.Ca = Ca (mmol/l)−((Alb(g/l)−30)×0.017) [27]. P-values were determined using Student’s unpaired, two tailed t-test. Skeletons of mice fixed in 10% formalin after terminal anaesthesia were subjected to digital radiography at 26kV for 3 seconds using a Faxitron MX-20 digital X-ray system (Faxitron X-ray Corporation, Lincolnshire, USA) [28].

### Mapping, DNA Sequencing and Genotyping

Genomic DNA was extracted from tail or auricular biopsies as described [29]. For genome-wide mapping, genomic DNA was amplified by PCR using a panel of 91 single nucleotide polymorphism (SNP) markers arranged in chromosome sets, and the products were analysed by pyrosequencing [30]. Individual exons and intron-exon boundaries of the *Xdh* gene were amplified from genomic DNA by PCR using gene-specific primers and Taq PCR Mastermix (Qiagen), and the PCR products sequenced using BigDye terminator reagents and ABI 3100 sequencer (Life Technologies, Carlsbad, USA) [31]. For genotyping, DNA was amplified by PCR using primers specific for exon 2 of *Xdh*, and PCR products were subject to restriction endonuclease digest using MseI (New England Biolabs). Digested products were analysed by agarose gel electrophoresis and images captured using a ChemiDoc XRS+ and Image Lab software (BioRad).

### Histological and Immunohistochemical Analysis

Dissected tissues were fixed in formalin, processed and embedded in paraffin blocks for histological and immunohistochemical analysis. Sections (3–4 µm) were stained with haematoxylin and eosin (H&E), von Kossa, periodic-acid Schiff, Masson’s trichrome and Congo red. For immunohistochemistry to detect XDH and COX-2 expression, sections were subject to heat-induced epitope retrieval in citrate buffer (pH6.0), followed by blocking in 10% goat or donkey serum, respectively, and incubation in rabbit anti-XDH (C-terminus) antibody (Santa Cruz, 1∶50) or goat anti-COX-2 antibody (Santa Cruz, 1∶200), respectively, at room temperature for 1 hour, and probed using the Envision+ (rabbit) kit (Dako) or donkey anti-goat horseradish peroxidase conjugated antibody (Jackson) and the Vector DAB kit, respectively. Nuclei were counterstained with haematoxylin. Stained sections were scanned and digital images captured using a Nanozoomer 2.0 virtual microscope (Hamamatsu Corporation, Japan). TUNEL staining was performed on sections using the ApopTag Fluorescein In Situ Apoptosis Detection kit (Millipore). Stained sections were mounted in VECTASHIELD® mounting medium containing DAPI (Vector laboratories, Peterborough, UK) to detect nuclei.
